# CCR7 and CD48 as Predicted Targets in Acute Rejection Related to M1 Macrophage after Pediatric Kidney Transplantation

**DOI:** 10.1155/2024/6908968

**Published:** 2024-06-24

**Authors:** Jie Zhang, Jun Pei, Chengjun Yu, Jin Luo, Yifan Hong, Yi Hua, Guanghui Wei

**Affiliations:** ^1^ Department of Urology Children's Hospital of Chongqing Medical University National Clinical Research Center for Child Health and Disorders Ministry of Education Key Laboratory of Child Development and Disorders, Chongqing, China; ^2^ Chongqing Key Laboratory of Structural Birth Defect and Reconstruction, Chongqing, China

## Abstract

**Background:**

Kidney transplantation (KT) is the best treatment for end-stage renal disease. Although long and short-term survival rates for the graft have improved significantly with the development of immunosuppressants, acute rejection (AR) remains a major risk factor attacking the graft and patients. The innate immune response plays an important role in rejection. Therefore, our objective is to determine the biomarkers of congenital immunity associated with AR after KT and provide support for future research.

**Materials and Methods:**

A differential expression genes (DEGs) analysis was performed based on the dataset GSE174020 from the NCBI gene Expression Synthesis Database (GEO) and then combined with the GSE5099 M1 macrophage-related gene identified in the Molecular Signatures Database. We then identified genes in DEGs associated with M1 macrophages defined as DEM1Gs and performed gene ontology (GO) and Kyoto Encyclopedia of Genomes (KEGG) enrichment analysis. Cibersort was used to analyze the immune cell infiltration during AR. At the same time, we used the protein–protein interaction (PPI) network and Cytoscape software to determine the key genes. Dataset, GSE14328 derived from pediatric patients, GSE138043 and GSE9493 derived from adult patients, were used to verify Hub genes. Additional verification was the rat KT model, which was used to perform HE staining, immunohistochemical staining, and Western Blot. Hub genes were searched in the HPA database to confirm their expression. Finally, we construct the interaction network of transcription factor (TF)-Hub genes and miRNA-Hub genes.

**Results:**

Compared to the normal group, 366 genes were upregulated, and 423 genes were downregulated in the AR group. Then, 106 genes related to M1 macrophages were found among these genes. GO and KEGG enrichment analysis showed that these genes are mainly involved in cytokine binding, antigen binding, NK cell-mediated cytotoxicity, activation of immune receptors and immune response, and activation of the inflammatory NF-*κ*B signaling pathway. Two Hub genes, namely CCR7 and CD48, were identified by PPI and Cytoscape analysis. They have been verified in external validation sets, originated from both pediatric patients and adult patients, and animal experiments. In the HPA database, CCR7 and CD48 are mainly expressed in T cells, B cells, macrophages, and tissues where these immune cells are distributed. In addition to immunoinfiltration, CD4+T, CD8+T, NK cells, NKT cells, and monocytes increased significantly in the AR group, which was highly consistent with the results of Hub gene screening. Finally, we predicted that 19 TFs and 32 miRNAs might interact with the Hub gene.

**Conclusions:**

Through a comprehensive bioinformatic analysis, our findings may provide predictive and therapeutic targets for AR after KT.

## 1. Introduction

Chronic kidney disease (CKD) will eventually develop into end-stage renal disease, with a morbidity of 10%, and is a global public health problem [1]. The epidemiology of CKD in children is 7–11.4/million people (2018), and the main cause is the kidney and urinary tract [2]. Long-term dialysis will seriously affect the growth and physical and mental health of children with uremia. Kidney transplantation (KT) is the best alternative treatment for children with uremia [3], but acute rejection (AR) is still one of the major and serious complications after KT, affecting the long-term survival of the graft and children [4]. The incidence of acute T-cell-mediated rejection confirmed by biopsy within 1 year after KT was 16%, and the incidence of subclinical rejection was 30% [5], and there was a higher proportion of persistent rejection in pediatrics relative to adult kidney transplant patients [5]. Even when reversed with high doses of hormones and powerful immunosuppressants, AR seriously affects the long-term survival of the transplanted kidney.

Previously, T lymphocytes were considered the main cell that infiltrates and destroys grafts; however, recent studies have shown that in addition to adaptive immune cells, innate immune cells also play an important role in graft rejection [6]. Normally, innate immunity is the first line of defense to resist potential pathogenic stimuli, such as infection and tumors. As an important component, macrophages have the ability to phagocytose and kill pathogenic microorganisms, recognize and present antigens, and mediate innate immunity [7]. Monocytes/macrophages kill target cells and regulate adaptive immunity mainly by promoting inflammation and antibody-dependent cytotoxicity [8, 9]. Vascular endothelial injury is one of the reasons for transplant organ dysfunction. Peripheral blood monocytes attach to the vascular endothelium, across the endothelial, after stimulation, migrate into tissues, and then develop into macrophages [10]. Peripheral blood monocytes are attracted to the kidney through MCP-1, CCL2, CX3CL1, MIP-1*α*, and so on [11, 12, 13]. The infiltrated macrophages in the graft are closely related to rejection and are always considered a characteristic of AR, and the total number of infiltrated macrophages in the grafts is associated with poor clinical outcomes and graft dysfunction [14, 15, 16, 17]. In severe rejection, the total of infiltrated macrophages accounted for 60% of the total infiltrated cells [18, 19].

Macrophages can differentiate into classically activated macrophages and alternatively activated macrophages. Classically activated macrophages, known as M1 macrophages, exert pro-inflammatory effects, while alternatively activated macrophages, known as M2 macrophages, counteract the effects of M1 macrophages and exert anti-inflammatory effects [20]. Therefore, M1 macrophages may be related to the occurrence of AR. Some research found that genetic signatures of inflammatory macrophage polarization have also been identified in rejection biopsy samples [21]. Macrophages can participate in graft rejection through multiple mechanisms [22]. Firstly, activated macrophages can directly phagocytose the graft cells. Secondly, M1 macrophages secrete a variety of pro-inflammatory mediators, such as IL-1*β*, IL-2, IL-6, IL-12, IL-18, IFN-*γ*, TNF-*α*, and nitric oxide (iNOS), which destroy microvessels, recruit leukocytes, and induce donor-specific cytotoxic reactions [23]. Infiltrated macrophages are also the main sources of reactive oxygen species and active nitrogen (RNS), which directly damage graft tissues and aggravate AR [24, 25, 26].

At present, prevalent immunosuppressive regimes are the main target of lymphocytes, such as calcineurin inhibitors (cyclosporine, tacrolimus), antiproliferative agents (Mycophenolate Mofetil), and mTOR inhibitors (Sirolimus, Everolimus) [27] which effectively target the T-cell-mediated rejection, while therapies targeting innate immune cells are not easy to obtain. Currently, macrophage-centered therapeutic strategies include macrophage depletion, inhibition of macrophage activation and migration, regulation of macrophage subpopulations, and promotion of immune regulation conducive to tolerance induction [6], but these projects still need a lot of research to explore.

In this study, we selected the renal biopsy sequencing data set of AR and nonrejection after KT in pediatric patients and then analyzed it with the dataset of M1 macrophages in order to find the molecular markers related to AR and M1 macrophages and to provide potential targets for prediction or intervention of rejection.

## 2. Materials and Methods

### 2.1. Dataset Sources and Processing

In this study, we took the words “AR of renal transplantation” as keywords and selected GSE174020 dataset [28] to analyze differentially expressed genes (DEGs) between the normal group and the AR group. The absolute value of log_2_fold > 1.0 and adj. *P*  < 0.05 were used as significance indicators.

The gene data set related to M1 macrophages GSE5099 [29] (*Supplementary 1*) was obtained from the Molecular Signatures Database (https://www.gsea-msigdb.org/gsea/msigdb/) and intersected with DEGs. The intersection was defined as differential genes related to macrophages M1 (DEM1Gs). To reveal the underlying biological functions and underlying mechanisms of DEM1Gs, we used Hiplot (https://hiplot.org), an online analysis tool for gene ontology (GO) and Kyoto Encyclopedia of Gene and Genes (KEGG) enrichment analysis of DEM1Gs as previous [30, 31].

### 2.2. Identification of Hub Genes Based on the Protein–Protein Interaction (PPI) Network and Cytoscape Analysis

To explore the interactions among DEM1Gs, the PPI network was constructed from the online biological resource database String (http://www.string-db.org/). Cytoscape software was used to visualize the PPI network of DEM1Gs, a confidence score of 0.4 as a cutoff value, and cytohubba was used to carry out Degree, MNC, MCC, Radiality, and Closeness algorithms; the top five genes were obtained by each algorithm for intersection. The intersection genes were thought to be the most valued M1 macrophage-related Hub genes for AR. Finally, the expression of Hub genes was statistically analyzed in the normal and AR group, where *P*  < 0.05 was considered statistically significant.

### 2.3. Verification of the Hub Gene

To verify whether the Hub genes have repeatability, we validate Hub genes both in pediatric and adult patients. The external validation sets, GSE143028 originated from pediatric patients [32], GSE138034 [33] and GSE9493 [34], originated from adult patients were selected in the GEO database to test the Hub genes, and verification of Hub genes was performed in animal models at the same time. The rat KT model was performed in our research. Twelve Sprague–Dawley rats (SD) rats aged 8–10 weeks were purchased from the Laboratory Animal Center of Chongqing Medical University (SYXK(YU) 2022-0016, Chongqing, China), and four Wistar rats aged 8–10 weeks were purchased from Beijing Vital River Laboratory Animal Technology Company (SCXK (jing) 2021-0006) and were housed at the Experimental Animal Center of Chongqing Medical University. KT was performed as previously described [35]. In summary, the donor rat was anesthetized with 2% pentobarbital sodium 40 mg/kg, and the left kidney was perfused with 5 ml of heparinized Ringer solution (25 u/ml) and removed. The donor's kidney was placed in a 1% heparinized saline solution at 4°C until the recipient rat was ready to receive the transplant. The recipient rat was anesthetized and underwent a left nephrectomy. The donor's kidney was placed in the posterior abdominal cavity of the recipient, and then we anastomosed the renal arteries, renal veins, and ureter of the donor and recipient. The right kidneys of the recipients were preserved, and no immunosuppressive agents were used after transplantation. The allo group setted Wistar rats as donors while SD rats as recipients (*n* = 4), and in the syn group, both the donor and the recipient were SD rats (*n* = 4), and rats between identical strains conform to the principle of randomization. During the experimental period, rats were raised with free water and food, and were sacrificed 7 days after transplantation, and all recipients survived to the time of sampling. Part of the kidney and spleen tissues were fixed with paraformaldehyde for pathological detection, such as HE staining and immunohistochemistry staining, while the rest were frozen with liquid nitrogen and stored at −80°C for protein detection.

### 2.4. Immunoinfiltration Analysis

To investigate related immune cell infiltration, we used Cibersort (http://cibersort.stanford.edu/) to calculate the percentage of immune cell samples in the GSE174020 data set, and the level of immune cell infiltration in the normal group and the AR group was compared with the “VioPlot” software package [36].

### 2.5. Construction of a Transcription Factors (TF)-Hub Prediction Network and miRNA-Hub Gene Network

NetworkAnalyst (https://www.networkanalyst.ca/NetworkAnalyst/) is a comprehensive expression spectrum data visualization tool, it can analyze the relationship of the target genes related to several types of biological molecules. We used the NetwoworkAnalyst tool for TF-Hub genes and miRNA-hub genes analysis. The corresponding results were inputted into the Cytoscape software to construct the visual regulatory network.

### 2.6. HE Staining and Immunohistochemical Staining

After the establishment of the animal model, to verify the successful establishment of the AR model, pathological tests were carried out in the experimental samples. Formaldehyde-fixed tissues were routinely embedded in paraffin, cut to 4 *µ*m, and HE staining was performed to determine the tissue structure destroy, and CD3 (proteintech, 176171-AP), CD4 (bioss, bs-0766R), CD8 (NOVUS, NBP-49045SS), and F4/80 (abclonal, A23788) immunohistochemical staining were used to determine the degree of immune cell infiltration. Immunohistochemical staining was performed using the EnVision two-step method and was performed strictly according to the instructions. The primary antibody was diluted to 1 : 200.

### 2.7. Western-Blot Detection

Kidney tissue was placed in RIPA dissolved buffer containing Protease Inhibitor Cocktail (MCE, HY-K000), fully homogenized, centrifuged at 4°C at 1.2 w rpm for 20 min, and the supernatant was taken and added to 5x buffer (MCE, WB2100) followed by western-blot detection. The experiment was carried out in strict accordance with the experimental procedure. TNF-*α* (huabio, ER65189), Bax (huabio, ET1603-34), and NGAL (abclonal, A3176) were detected to determine the protein expression of tissue injury, and then the Hub gene protein was detected. Each protein expression was normalized by *β*-actin (abways, ab0039) or GAPDH (abways, ab0037), and semiquantitative statistical analysis was performed by Image Lab6.0.1.

### 2.8. Statistical Analysis

GraphPad Prism 9.5.0 was used for data statistics and analysis. All statistical data was tested using a positive distribution test and a variance homogeneity test, and then Student's *T* test or nonparametric test was performed to compare the differences between studying groups.  ^*∗∗∗∗*^ Represents *P*  < 0.0001,  ^*∗∗∗*^ represents *P*  < 0.0001,  ^*∗∗*^ represents *P*  < 0.01,  ^*∗*^ represents *P*  < 0.05, *P*  < 0.05 indicates a statistically significant difference, and ns represents no significance. Adobe Illustrator 27.0.0 and Adobe Photoshop 23.5.0 were used for image collation.

## 3. Results

### 3.1. Identification and Enrichment Analysis of DEM1Gs

**Figure 1:**
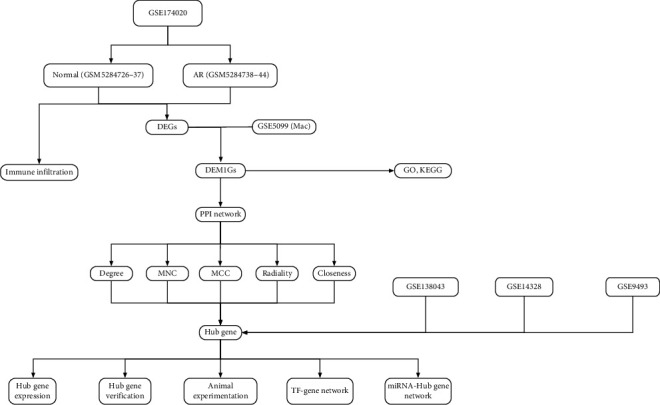
Research flowchart in this study.

**Figure 2:**
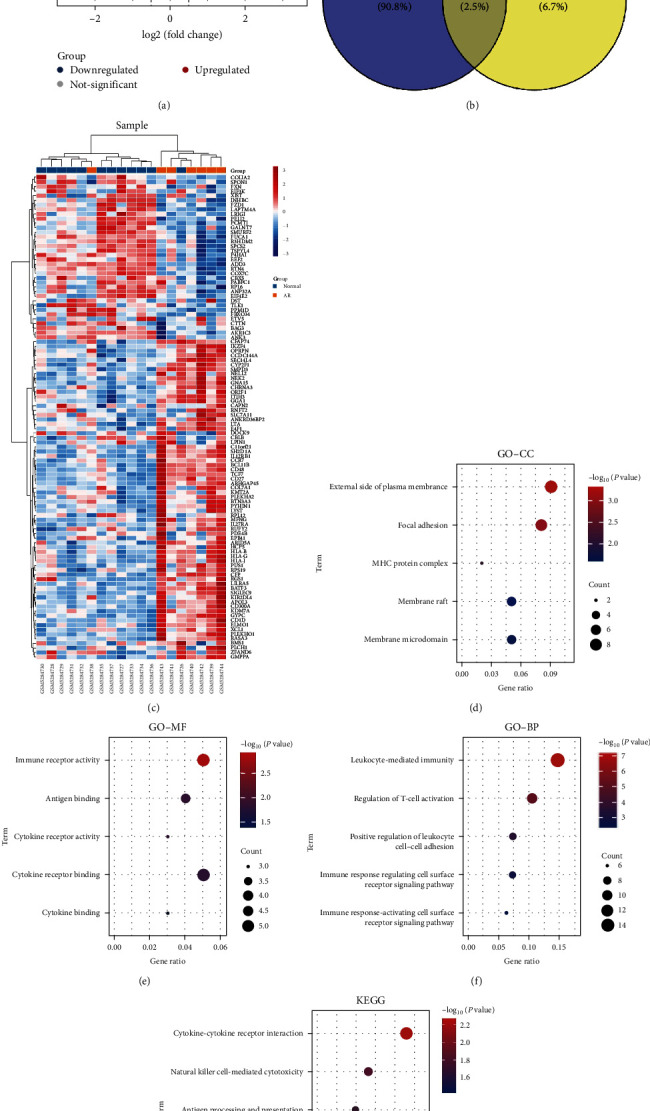
Identification and enrichment analysis of DEM1Gs: (a) volcano map of DEGs in dataset GSE174020; (b) Veen plot of the intersection of DEGs and M1 macrophage-related genes (GSE5099); (c) heat map of DEM1Gs; (d–f) the GO enrichment analysis of DEM1Gs; (g) KEGG enrichment analysis of DEM1Gs.

In this study, the flowchart of this study is shown in (Figure 1). Select the GSE174020 dataset (*Supplementary 2*) in the GEO database, the differential genes between the normal group and the AR group were analyzed (log_2_fold absolute value > 1.0 and adj. *P*  < 0.05), among which 366 genes were upregulated, and 423 genes were downregulated in the AR group. The volcano map showed the distribution of these DEGs (Figure 2(a)), and the heat map showed that differential gene analysis can distinguish between nonrejection and rejection (Figure 2(b)). DEGs were intersected with M1 macrophage-related genes (dataset GSE5099), and 106 AR associated with M1 macrophage-related genes (DEM1Gs) were obtained (Figure 2(c)). GO analysis showed that, in terms of cellular component (CC), these genes are located mainly on cell membranes and vesicles (Figure 2(d)). For molecular function (MF), DEGs are mainly involved in cytokine binding, antigen presentation, and activation of immune receptors (Figure 2(e)). The biological process (BP) mainly regulates leucocyte-mediated immune responses, T cell activation, and activation of the cell surface receptor pathway (Figure 2(f)). KEGG enrichment analysis showed that DEM1Gs mainly enriched 12 pathways, including AR-related biological processes, such as cytokine interaction with cytokine receptors, NK cell-mediated cytotoxicity, antigen presentation, allograft rejection, and activation of the classical inflammatory pathway NF-*κ*B (Figure 2(g)). These differential genes are indeed associated with rejection-related immunity.

### 3.2. Identification of the Hub Gene Based on the PPI Network and Cytoscape

**Figure 3:**
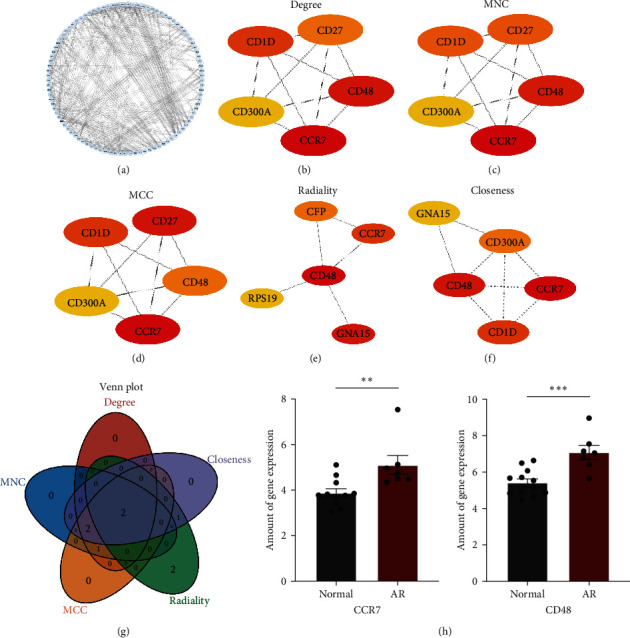
Identification and verification of the Hub gene: (a) PPI network of DEM1Gs; (b–f) the five algorithms of Cytoscape cytohubba, namely Degree, MNC, MCC, Rediality, and Closeness; (g) intersection gene after passing the algorithm, namely Hub gene; (h) validation of Hub gene in the GSE174020 dataset.  ^*∗∗∗*^ Represents *P*  < 0.001,  ^*∗∗*^ represents *P*  < 0.01.

We inputted the 106 DEM1Gs obtained above into the String database, screened the proteins that interacted with them, and then inputted them into Cytoscape software to construct the PPI network (Figure 3(a)). Then, the top five results of Degree, MNC, MCC, Radiality, and Closeness algorithms were interpreted for intersection analysis (Figures 3(b), 3(c), 3(d), 3(e), and 3(f)). Eventually, the two most vital Hub genes were identified, namely CCR7 and CD48 (Figure 3(g)). These two Hub genes are considered to be the most relevant genes for M1 macrophages in AR after KT. The protein expression levels of CCR7 and CD48 were statistically analyzed, and it was found that the expression levels of CCR7 and CD48 in patients with AR were higher than those of the normal group, and the difference was statistically significant (Figure 3(h)).

### 3.3. Verification of Hub Genes

**Figure 4:**
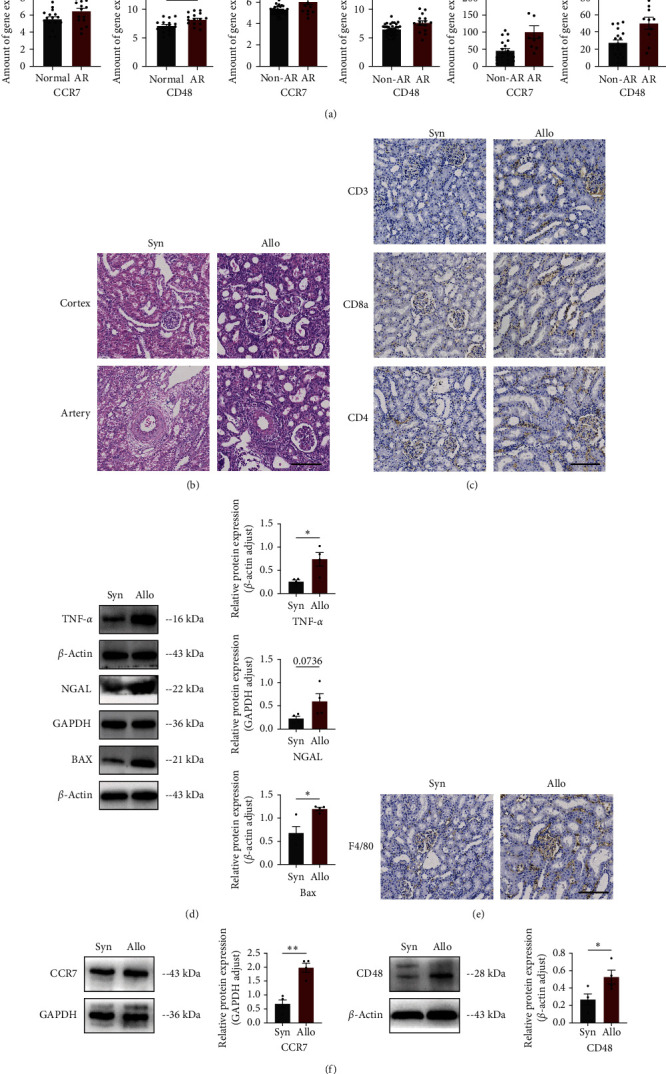
Verification of the Hub gene in external datasets and animal models: (a) validation of the Hub gene in the external pediatric patients dataset GSE14328, and adults patient datasets, GSE138043 and GSE9493; (b) HE staining of the rat graft; (c) immunohistochemical staining of CD3, CD4, and CD8 T cells; (d) western blot results and semi-quantitative statistical analysis of the damage indices Bax, NGAL, and TNF-*α*; (e) immunohistochemical staining of F4/80 macrophages; (f) western blot of Hub gene expression.  ^*∗∗∗*^ Represents *P*  < 0.001,  ^*∗∗*^ represents *P*  < 0.01,  ^*∗*^ represents *P*  < 0.05, ns represents *P*  > 0.05; bar is 50 *μ*m.

To further verify the reliability and repeatability of Hub genes, statistical analysis of CCR7 and CD48 expression was performed on external validation sets, GSE14328, GSE138043, and GSE9493, and it was found that CCR7 and CD48 had the same trend in the training set, both were elevated in AR after KT (Figure 4(a)), consistent with previous findings. It is worth emphasizing again that the external validation sets are separately derived from pediatrics and adults.

In addition, CCR7 and CD48 were validated in animal models. The first was to prove whether the AR model was successfully established. HE staining showed that there was a large number of hyperchromatic nuclear cell infiltration in the interstitium and perivascular area of the renal tubule in the allo group compared to the syn group (Figure 4(b)), while immunohistochemical staining showed infiltration of CD3, CD4, and CD8a T cells (Figure 4(c)) and increased macrophage infiltration (Figure 4(e)). These evidences revealed that the graft was suffering rejected, as well as some other indexes of damage and inflammation by western blot (Figure 4(d)). Furthermore, western blot experiments showed that the protein expressions of CCR7 and CD48 in the rejected graft were higher than those of the syn group (Figure 4(f)), which was consistent with the results of previous studies. Therefore, we suggested that CCR7 and CD48 are associated with AR related to M1 macrophages after KT.

**Figure 5:**
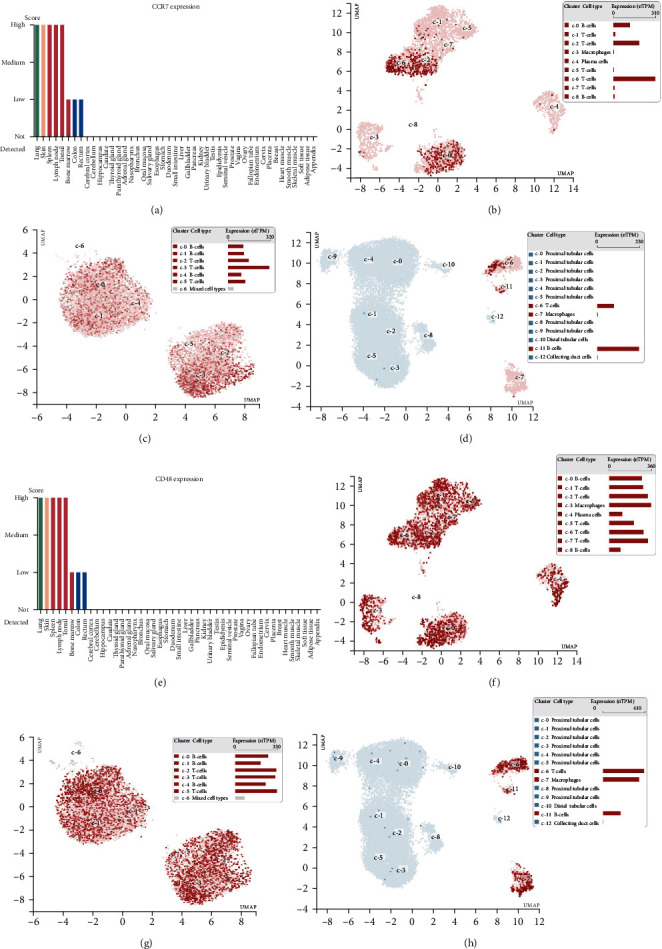
Distribution and expression of the Hub gene in the human body: (a) distribution of CCR7 expression in the human body; (b) single-cell sequencing of the spleen, cell location of CCR7; (c) single-cell sequencing of lymph nodes and cell localization of CCR7; (d) single-cell sequencing of kidney, cell localization of CCR7; (e) distribution of CD48 expression in the human body; (f) single-cell sequencing of the spleen, cell localization of CD48; (g) single-cell sequencing of lymph nodes, cell location of CD48; (h) single-cell sequencing of kidneys, cell location of CD48.

To explore the expression of hub genes in the body, we searched for CCR7 and CD48 in the HPA database. CCR7 and CD48 were found to be expressed mainly in immune organs, other than the lung, skin, and intestine, such as the spleen, lymph nodes, tonsils, and other immune tissues (Figures 5(a) and 5(e)). Single-cell sequencing of the spleen and lymph nodes showed that CCR7 and CD48 were mainly located on T cells and macrophages (Figures 5(b), 5(c), 5(f), and 5(g). Expressions of CCR7 and CD48 were also detected in kidney single-cell sequencing, but they were not in renal tissue cells but in some resident immune cells (Figures 5(d) and 5(h). These results indicated that immune cells express CCR7 and CD48 mainly, especially T cells and macrophages.

### 3.4. Analysis of Immune Infiltration

**Figure 6:**
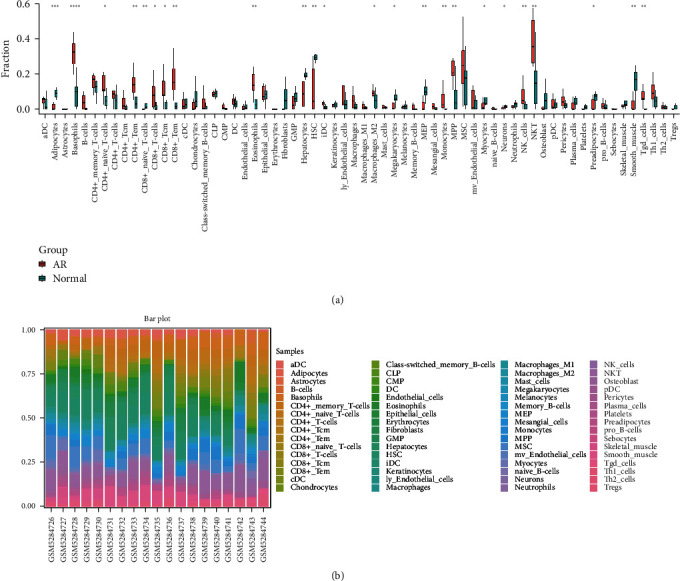
Analysis of immune cell infiltration: (a) analysis of the difference in immune cell infiltration in the normal group graft and the AR group in the GSE174020 training set; (b) types of immune cell infiltration in each sample in the normal and AR groups.  ^*∗∗∗∗*^ Represents *P*  < 0.0001,  ^*∗∗∗*^ represents *P*  < 0.001,  ^*∗∗*^ represents *P*  < 0.01,  ^*∗*^ represents *P*  < 0.05.

According to GO and KEGG enrichment analysis, it was found that DEGs were closely associated with immune cells. Immunoinfiltration analysis was performed in the normal group and the AR group of the GSE174020 training set and found that CD4+T, CD8+T cells, NK cells, NKT cells, and monocytes were significantly different between the two groups (Figure 6), these cells being the most concerned in graft rejection. Almost similar to the findings above, CCR7 and CD48 interact with these immune cells.

### 3.5. Construction of the TF-Hub Gene Network and miRNA-Hub Gene Network

**Figure 7:**
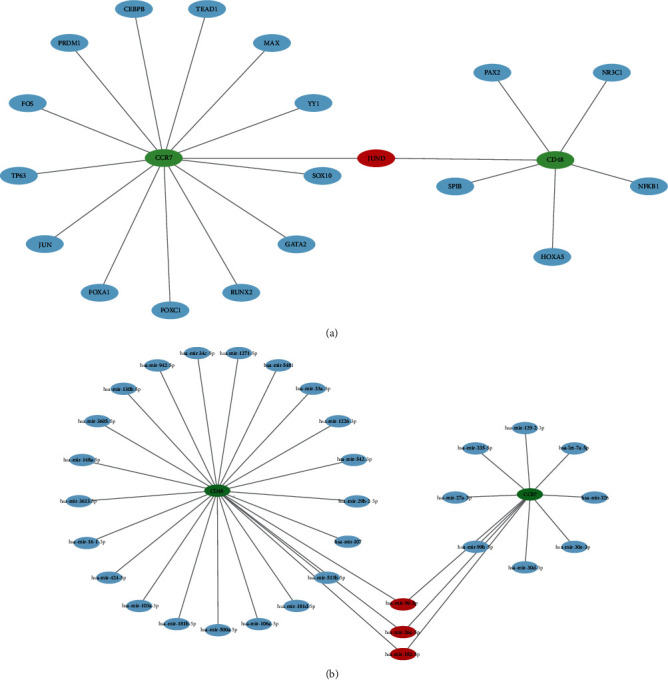
Construction of TF-hub genes and miRNA-hub genes network: (a) TFs regulatory network of hub genes; (b) MiRNAs regulatory network of hub genes. Green represents the Hub genes, blue represents TFs, and red represents TFs jointly expressed by two Hub genes.

To understand the regulatory role of the Hub gene in AR, we explored related TFs and miRNAs associated with Hub genes. A total of 19 TFs related to the Hub gene were identified, among which 14 TFs regulated CCR7, 6 TFs regulated CD48, and one TF could regulate both CCR7 and CD48 (Figure 7). We also predicted miRNAs that interact with the Hub genes, and 32 miRNAs were identified to interact with them, of which 24 miRNAs were associated with CCR7, 11 miRNAs were associated with CD48, while 3 miRNAs were related to both Hub genes (Figure 7). Similarly, these results were inputted into Cytoscape to construct the visual network. These TFs and miRNAs provide support for our research on the potential roles and mechanisms of CCR7 and CD48.

## 4. Discussion

With the development of immunotherapy, the incidence of AR after KT has improved significantly [27]. However, there is still a proportion of patients who suffer from AR, especially in pediatric patients, affecting the outcomes of KT. Therefore, early recognition and intervention of AR is crucial for patients after KT. Currently, the diagnosis of rejection still relies on renal biopsy; therefore, we also chose renal biopsy sequence data for our study. By bioinformatics analysis, CCR7 and CD48 were found to be the key genes significantly associated with AR related to the M1 macrophages, which were validated by external datasets and animal experiments.

In this study, we selected the renal biopsy sequencing of pediatric KT patients (GSE174020) for the comparison of rejection versus nonrejection, with a very large number of genes involved (DEGs), as visualized by the volcano plots, and illustrating that rejection occurs as a complex and intertwined process. Our main focus was on the association of innate immunity with AR, particularly inflammatory macrophages and M1 macrophages. Therefore, we chose the GSE5099, M1 macrophage-related gene dataset, to intersect with DEGs, identified106 related genes (DEM1Gs), and performed heatmap analysis on DEM1Gs and found that these genes do distinguish between rejection and nonrejection, proving that these genes are AR genes related to M1 macrophage. To explore the biological functions of DEM1Gs, we found that these 106 differential genes were mainly involved in cell–cell adhesion, activation of immune cells, and regulation of leukocyte immune response by GO enrichment analysis. KEGG enrichment analysis revealed that these genes were significantly associated with AR-associated pathways such as antigen presentation, NK cell-mediated cytotoxicity, and graft rejection, as well as the inflammatory pathway, NF-*κ*B, activation. These results could demonstrate that M1 macrophage-mediated rejection is mainly about antigen presentation, regulation, activation of other immune cells, and inflammation. Conde et al. [37] demonstrated that macrophages, as important antigen-presenting cells, activate T cells and contribute to their differentiation and proliferation. Kloc et al. [38] revealed that macrophages secrete a variety of cytokines that result in graft rejection, such as IL-6 and IL-1*β*, and Krausgruber et al. [39] similarly demonstrated this. Thus, it can be seen that the immunological role of innate immunity after KT should not be ignored.

In this study, the PPI network and the cytohubba algorithm were used to identify key genes, and finally, CCR7 and CD48 were identified and considered to be the most diagnostic values. The expression of CCR7 and CD48 protein in the AR group in the training set is significantly higher than in the normal group. In the external validation set GSE14328, a dataset derived from pediatric patients, CCR7 and CD48 expression levels were higher in the AR group compared to the normal group. In addition, in adult patient datasets, GSE138043 and GSE9493, the expression of CCR7 and CD48 were consistent with that of the training set, with statistical differences as well. As summarized by Dharnidharka [40], younger recipients seem to have better outcomes after KT, because of the more naïve immune system compared to adults, including the low expression of CD40L on T cells [41], higher level of Th2 cells [42] and tolerogenic dendritic-cell subset [43]. However, it is worth noting that the Hub genes, CCR7 and CD48, identified from the pediatric patient's dataset, were equally suitable for adult patients, and the expression of the hub genes elevated when they suffered from AR after KT. This seems to predict that the rejection mechanisms of the kidney graft are generally similar. Based on this result, we can conclude that CCR7 and CD48 are molecules capable of serving as predictors and diagnostics of AR, although there are immune system differences between children and adults.

Similarly, the validation of CCR7 and CD48 expressions of animal models showed consistent results, demonstrating the reproducibility of the Hub genes. First of all, we performed experiments to verify the successful establishment of AR models related to non-rejection. HE staining, compared to the syn group, showed a large infiltration of immune cells in the tubular interstitium and perivascular area in the allo group, manifested as glomerulonephritis, tubulonephritis, and arteritis. To confirm the occurrence of rejection more clearly, we also performed immunohistochemical staining of T cells and macrophages and found that in the allo group, there were a variety of T cells clustering around the glomeruli and tubular interstitium, including CD4+ T and CD8+ T cells, and these activated T cells were the main cells in the rejection reaction [44]. F4/80, the macrophage marker, was also detected and was similarly heavily aggregated around the tubules in the allo group. In addition to some injury markers, such as bax and NGAL, protein expression of the inflammatory marker tnf-*α* was significantly higher in the allo group than those in the syn group. These experimental animal results proved the important role of immune cells, inflammatory factors, and some proteins in the rejection reaction process, and it is just as Cheng et al. [45] described that IRI could promote the expression of NGAL in grafts, resulting in increased macrophage infiltration in mouse cardiac grafts. Niederkorn et al. [46] found that activated mononuclear phagocytes secreted interleukin (IL)-1*β*, which mediates the migration of antigen-presenting cells into allografts and triggers allograft rejection. Combined with the previous detection of CCR7 and CD48 expression, it was therefore concluded that Hub genes could serve as relevant markers in AR.

In addition, we searched for the expression of CCR7 and CD48 in the HPA database and found that they were abundantly expressed in immune tissues of the spleen, lymph nodes, and tonsils in addition to skin and lung tissues, and in single-cell sequencing of the spleen, we found that CCR7 and CD48 were abundantly expressed on T and B cells and macrophages. In single-cell sequencing of lymph nodes, only T and B cells were detected, which may be due to the fact that the lymph nodes are mainly a congregation of T and B cells. Additionally, CCR7 and CD48 were also found to be expressed in T and B cells and macrophages in single-cell kidney sequencing. Thus, it can be seen that CCR7 and CD48 are highly relevant to the biological functions of T, B, and macrophage cells, which are the key targets of concern in the KT.

CCR7 is a G protein-coupled receptor that is expressed primarily on the surface of B cells, DCs, and T cells, and its ligands are CCL19 and CCL21 [47]. We also found it in macrophages. Han et al. found that CCL21 is mainly produced by stromal cells and lymphatic endothelial cells; its expression is significantly increased under inflammatory conditions and in autoimmune diseases. CCL21 promotes the adhesion and migration of a variety of immune cells, including T lymphocytes, macrophages, and neutrophils, by binding with high affinity to CCR7. It also promotes the proliferation of bone marrow DCs by phosphorylating NF-*κ*B p65 to participate in the regulation of inflammatory immune responses [48]. Furthermore, the CCL21/CCR7 axis has been shown to have a proliferative effect on CD4+ CD8+ T cells, mesangial cells, etc. [49]. Cuesta-Mateos et al. [50] used anti-CCR7 mAb to alleviate graft-versus-host disease (GVHD). Similarly, Varlet et al. [51] treated GVHD by depleting CD4+CCR7 T cells. Lin et al. [52] found that inhibition of the migratory effects of CCL21/CCR7 alleviated chronic rejection.

CD48 (SLAMF2) is involved in the adhesion activation of immune cells, and CD48 is expressed in almost all nucleated hematopoietic cells [53]. The functional outcome of CD48-2B4 binding may vary depending on the cell pair. The interaction of CD48 and 2B4 in NK cells induces the expansion and activation of NK cells and T cells [54, 55, 56]. Lissina et al. [57] reduced CD8+ T-cell cytotoxicity and cell functional effectors by inhibiting the binding of CD244 to CD48. Zou et al. [58] also demonstrated that CD48 plays a very important role in inflammation and tumor immunity. Qin et al. [59] found that the use of anti-CD2 combined with anti-CD48 mAb prolonged the lifespan of allogeneic cardiac transplants. McArdel et al. [60] found that anti-CD48 mAb attenuated experimental autoimmune encephalomyelitis by limiting the number of pathogenic CD4+ T cells. Through previous studies, we could find that CCR7 and CD48 are important targets of action in inflammation and immunity and would be potential targets for preventing or mitigating rejection after KT.

According to the immune infiltration analysis, we found that the cellular composition varied significantly. We mainly found that immune cells such as CD4+ T, CD8+ T, NK cells, NKT cells, and monocytes were increased in the AR group compared to the normal group, once again suggesting that infiltration of immune cells plays a much more significant role in AR of the allograft. Einecke et al. [61] described macrophage activation in mouse allogeneic dynamics in KT, and these allografts showed an increase in macrophage activation-related transcripts, including allograft inflammatory factor 1 (Aif1), nitric oxide synthase 2 (Nos2) and TNF-*α* on day 1 after transplantation, and an increase in macrophages within the grafts on 2 days posttransplantation, followed by a gradual increase. Therefore, most of the clinical measures to counteract rejection are to inhibit immune cells proliferation and activation, such as the preoperative application of ATG [62] and postoperative administration of immunosuppressive agents [63].

TFs are the major regulators of biological processes that modulate the expression of multiple target genes and create feedback regulation. Many genes are already altered during the initial stages of disease development, including TFs. Kobayashi et al. [64] found that the TF c-JUN was activated in AR [64]. Wu et al. [65] found that activation of TFs, such as NF-*κ*B, STAT1, and STAT 3, was associated with AR. Sui et al.'s [66] study found that a variety of TFs are closely related to the occurrence of AR. These studies and our TFs selected were partially overlapped, including MAX, GATA, NF-*κ*B, etc., and were consistent with our findings. Sui et al. [67] also investigated the regulation of AR by miRNAs; for example, has-mir-326 was regulated by TFs such as AP1, AP4, P53, etc., which may provide predictive clues to AR. However, studies targeting TFs and miRNAs in AR after KT are still limited and need development. However, we can speculate that CCR7 and CD48 interact with various TFs and miRNAs and are involved in AR regulation.

In this study, we used PPI Network and Cytoscape software to identify biomarkers associated with AR related to the M1 macrophage. In addition, an immune infiltration analysis was performed, as well as animal experiments for validation. However, the present study still has limitations. First, we based our study on previously published datasets, and different conclusions may be reached depending on different analysis methods or perspectives, the use of different tools, and their versions. Second, we lacked clinical samples for validation, which is unrealistic for clinical translation. We did not sequence animal samples, but this is our subsequent research on KT. Finally, this study still needs more experiments to demonstrate the mechanism of CCR7 and CD48 in AR related to macrophages after KT.

## 5. Conclusions

In conclusion, we conducted a comprehensive bioinformatics analysis of AR after renal transplantation and found that the Hub genes CCR7 and CD48 play a key role in the regulation of immune cells and are involved in the development of AR after renal transplantation. In addition, several TFs and miRNAs that may regulate the Hub genes were revealed, providing the possibility of exploring the mechanisms in more depth. The findings of this study may serve as predictors and potential therapeutic targets of AR after KT and provide a certain basis preliminary basis for later studies.

## Data Availability

The data sets presented in this study can be found in online repositories. The original data of the animal experiments can be found in the Supplementary Materials section (*Supplementary 3*).

## Supplementary Materials

Supplementary 1 Macrophages polarization related genes data set.

Supplementary 2 Original data set analyzed in this study.

Supplementary 3 Animal experimental raw data.

Supplementary 4 The Animal Research: reporting In Vivo Experiments (ARRIVE) guidelines.
