# Supplementary material for: CCR7 and CD48 as Predicted Targets in Acute Rejection Related to M1 Macrophage after Pediatric Kidney Transplantation
Source: J Immunol Res. 2024 Jun 24;2024:6908968. doi: 10.1155/2024/6908968 (PMC11217580; doi:10.1155/2024/6908968)

The datasets used in this study are available in public databases, and they were reflected in the manuscript.The following is the raw data of the animal experiment portion in this study.

TNF-α

group information: syn\allo

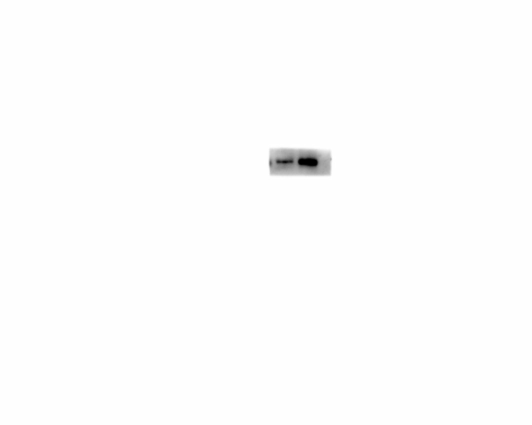

β-actin

group information: syn\allo

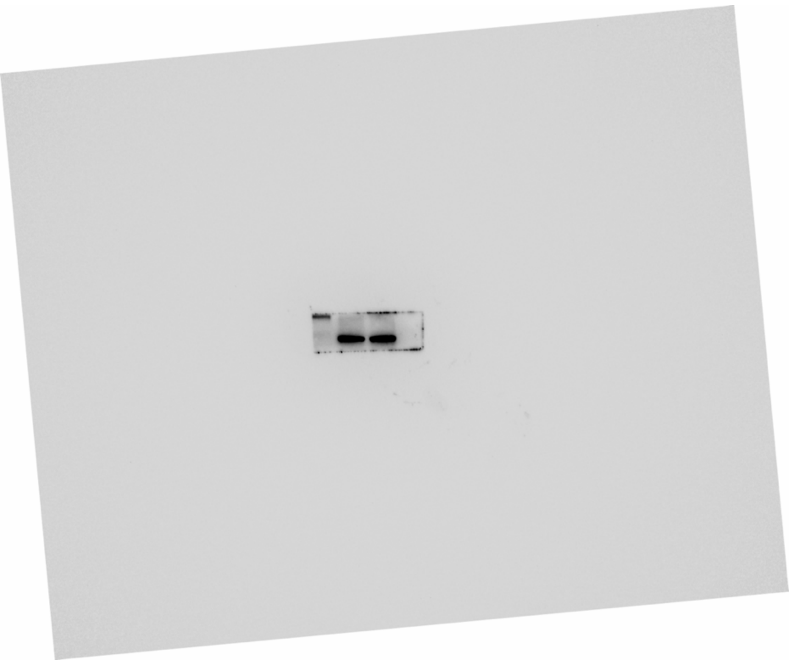

NGAL

group information: syn\allo

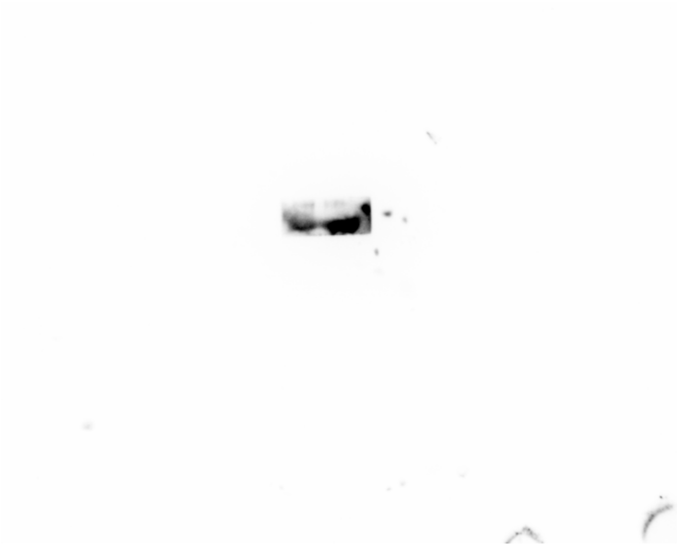

GAPDH

group information: syn\allo

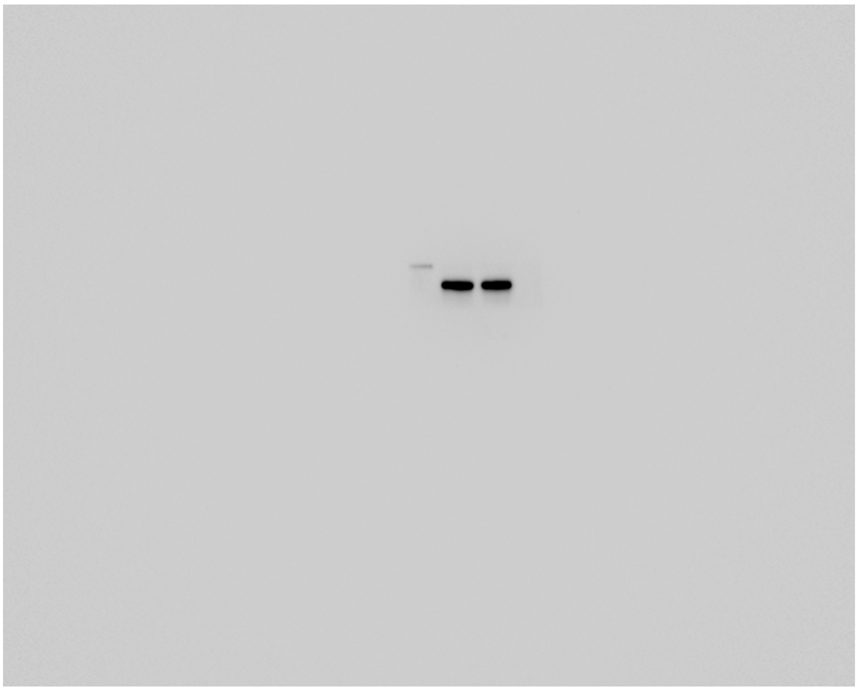

Bax

group information: syn\allo

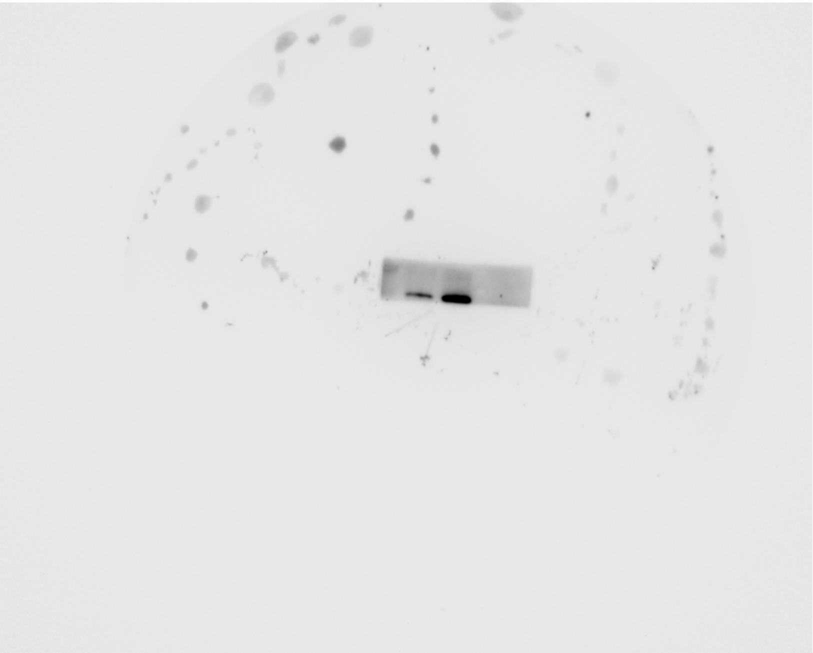

β-actin

group information: syn\allo

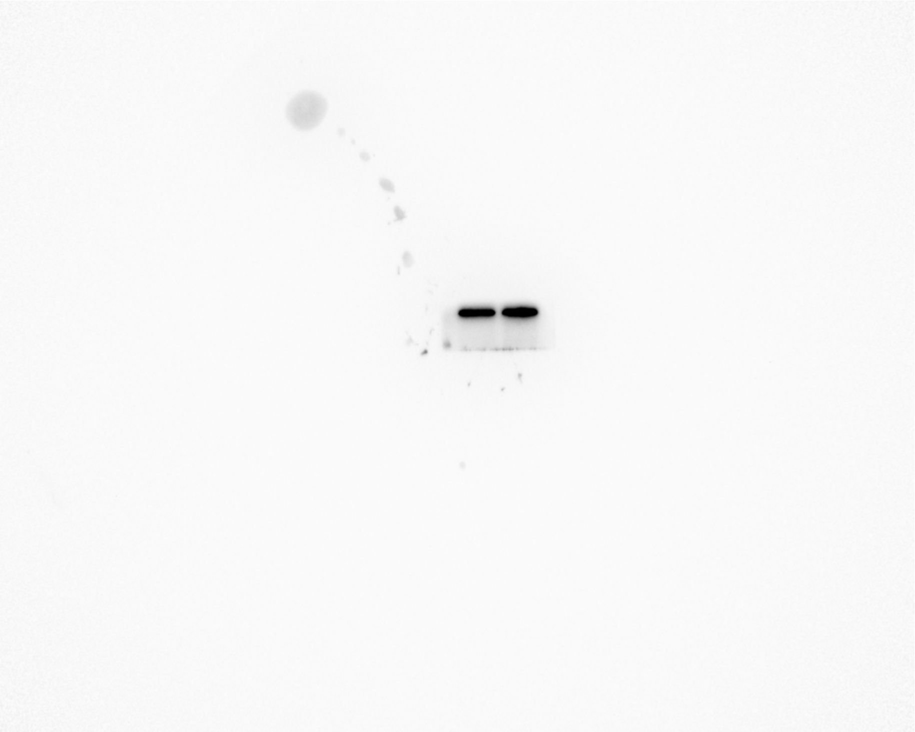

CCR7

group information: syn\allo, syn\allo, syn\allo, syn\allo

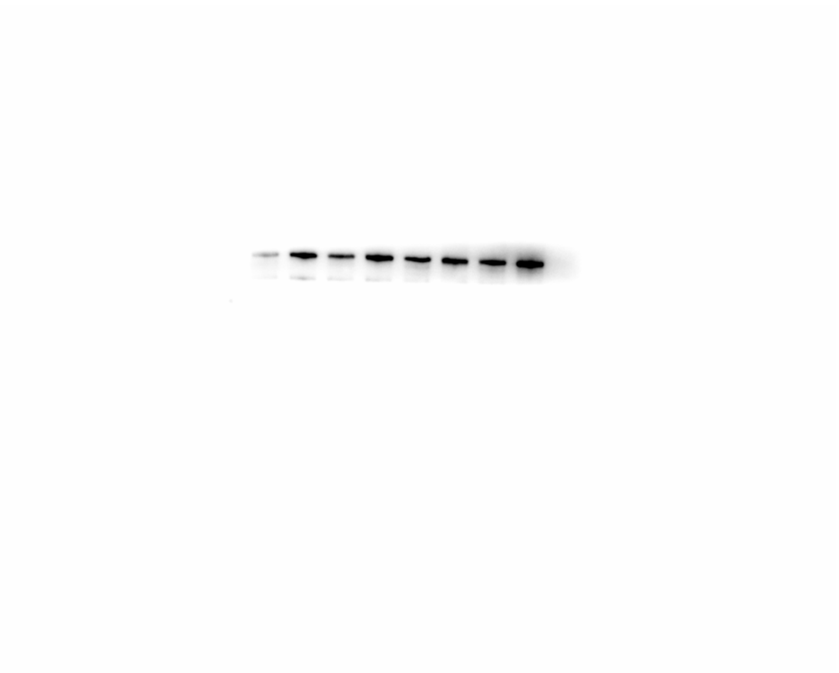

GAPDH

group information: syn\allo, syn\allo, syn\allo, syn\allo

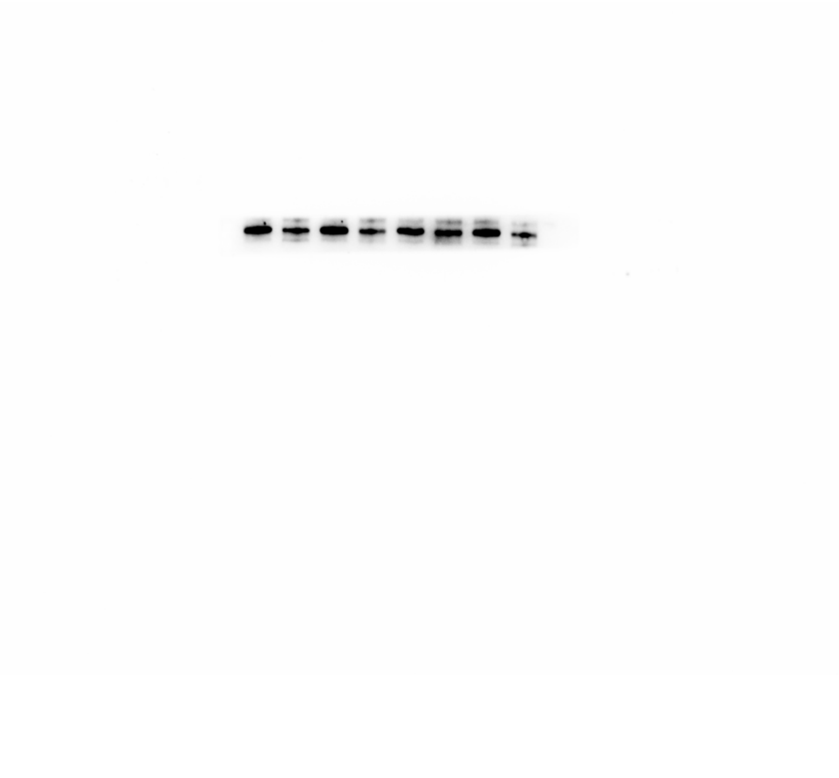

CD48

group information: syn\allo, syn\allo, syn\allo, syn\allo

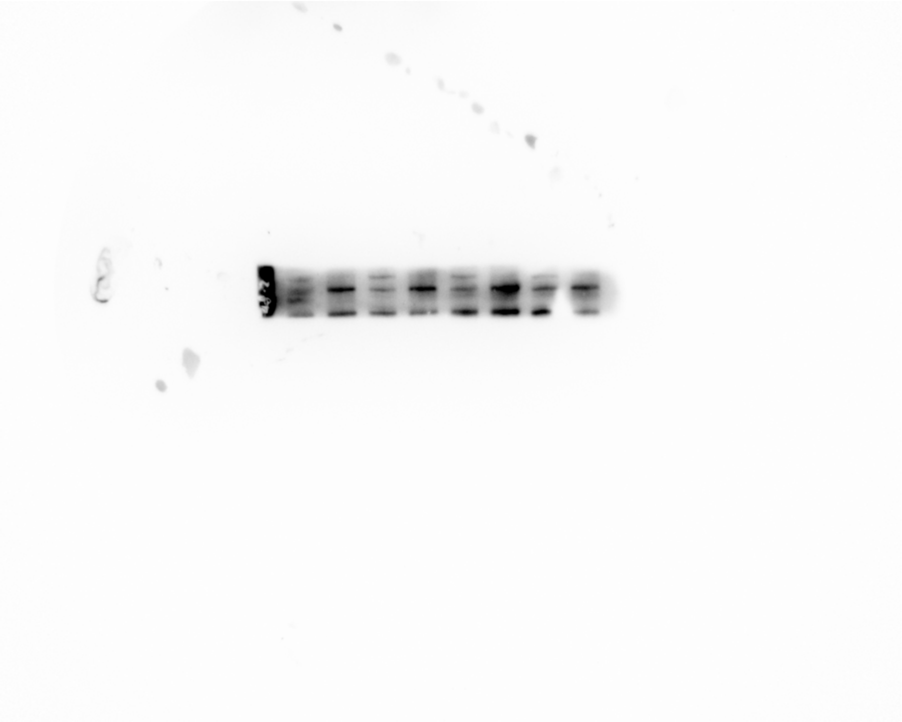

β-actin

group information: syn\allo, syn\allo, syn\allo, syn\allo

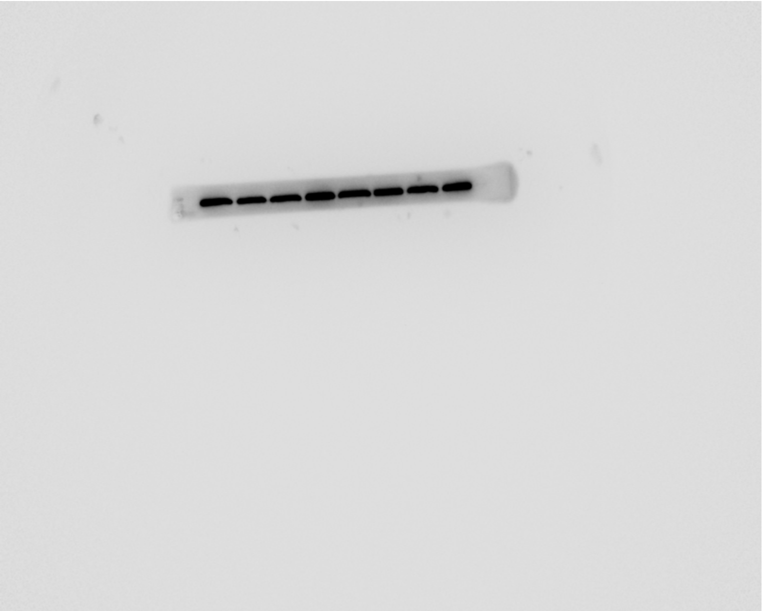

external datasets of GSE138043

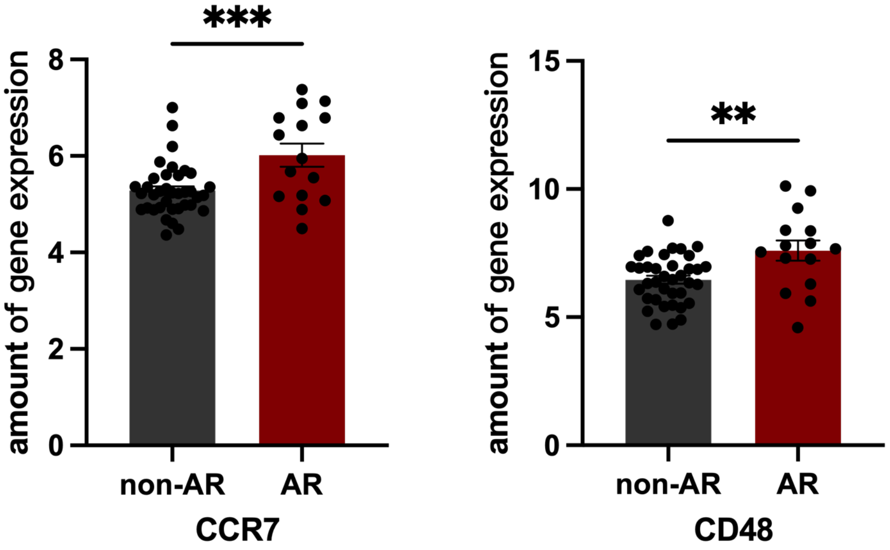

external datasets of GSE9493

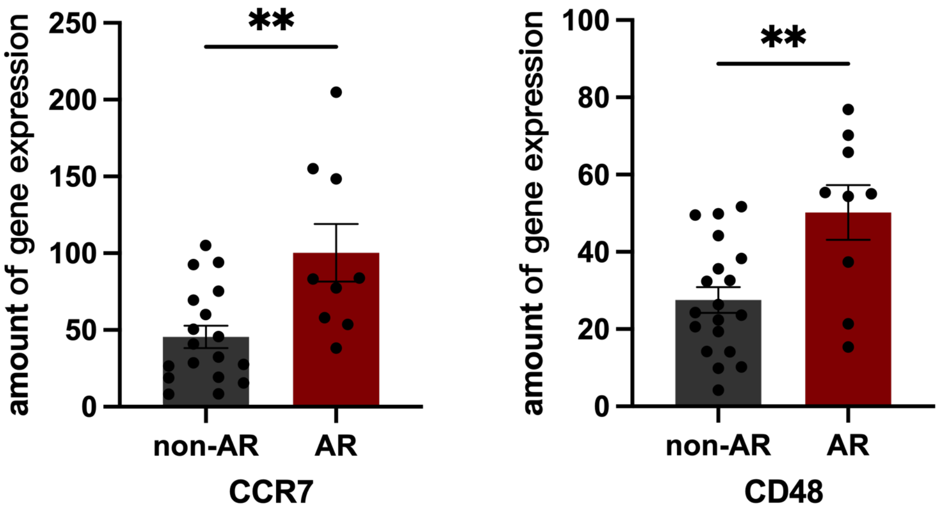

HE

Group information: syn\allo, syn\allo

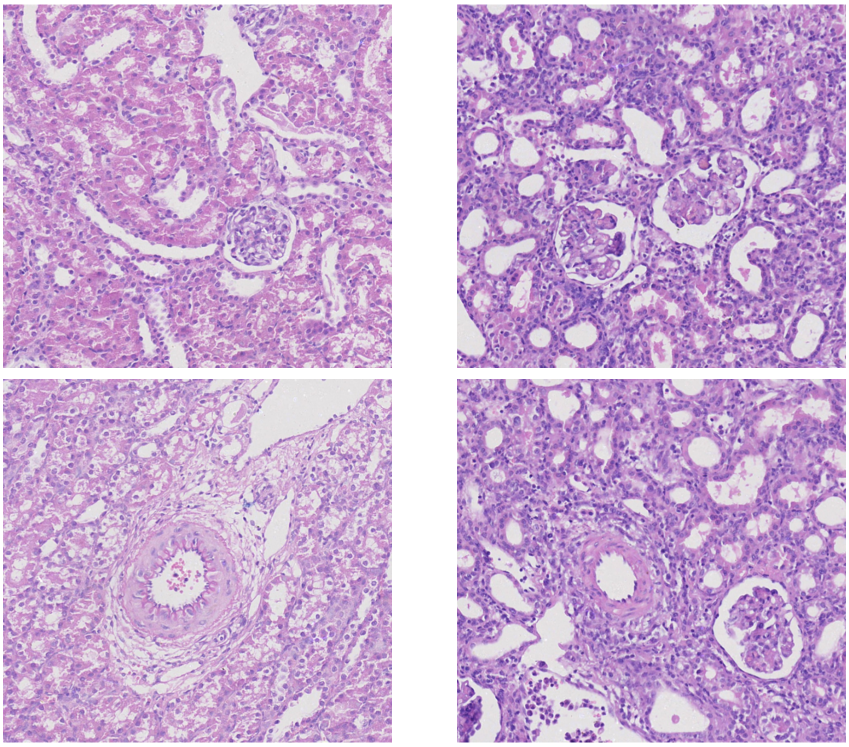

Immunohistochemical staining of CD3

Group information: syn\allo

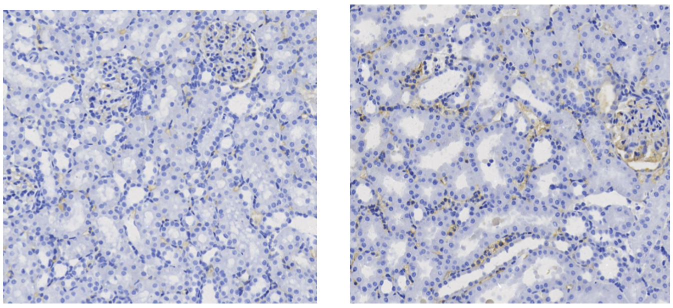

Immunohistochemical staining of CD4

Group information: syn\allo

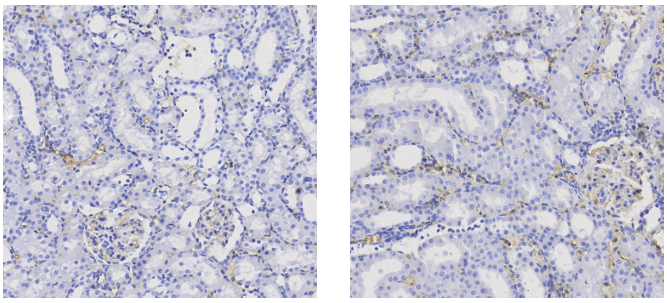

Immunohistochemical staining of CD8a

Group information: syn\allo

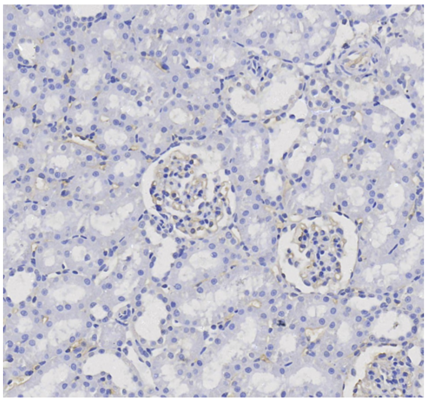

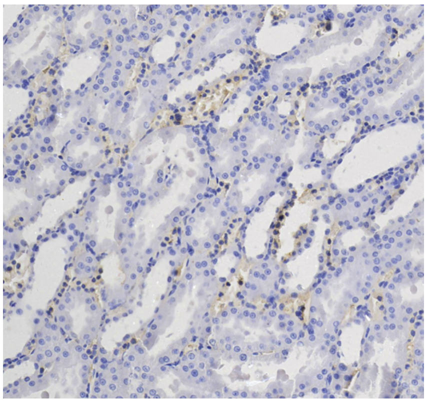

Immunohistochemical staining of F4\80

Group information: syn\allo

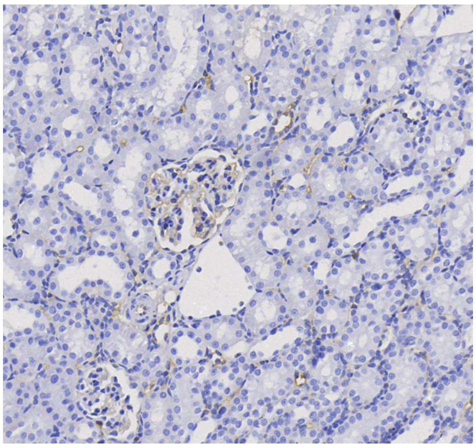

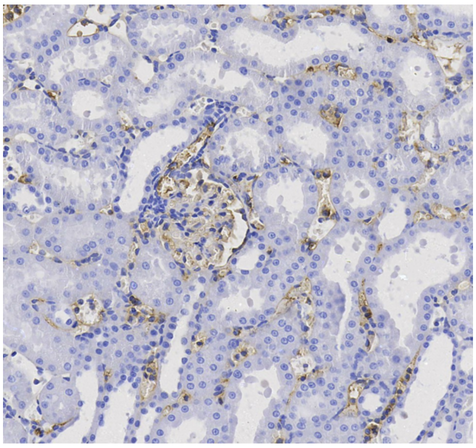

Supplement: Supplementary 2 — Original data set analyzed in this study. [file 6908968.f2.pdf]
